# Identification of a prognostic risk-scoring model and risk signatures based on glycosylation-associated cluster in breast cancer

**DOI:** 10.3389/fgene.2022.960567

**Published:** 2022-10-20

**Authors:** Shengnan Gao, Xinjie Wu, Xiaoying Lou, Wei Cui

**Affiliations:** ^1^ Department of Clinical Laboratory, National Cancer Center/National Clinical Research Center for Cancer/ State Key Laboratory of Molecular Oncology, Cancer Hospital, Chinese Academy of Medical Sciences and Peking Union Medical College, Beijing, China; ^2^ Peking University China-Japan Friendship School of Clinical Medicine, Beijing, China; ^3^ Department of Orthopedic Surgery, China-Japan Friendship Hospital, Beijing, China; ^4^ Department of Molecular Medicine and Surgery, Center for Molecular Medicine, Karolinska Institutet, Stockholm, Sweden

**Keywords:** breast cancer, glycosylation, prognosis, subtype, biomarkers, immune

## Abstract

Breast cancer is a heterogeneous disease whose subtypes represent different histological origins, prognoses, and therapeutic sensitivity. But there remains a strong need for more specific biomarkers and broader alternatives for personalized treatment. Our study classified breast cancer samples from The Cancer Genome Atlas (TCGA) into three groups based on glycosylation-associated genes and then identified differentially expressed genes under different glycosylation patterns to construct a prognostic model. The final prognostic model containing 23 key molecules achieved exciting performance both in the TCGA training set and testing set GSE42568 and GSE58812. The risk score also showed a significant difference in predicting overall clinical survival and immune infiltration analysis. This work helped us to understand the heterogeneity of breast cancer from another perspective and indicated that the identification of risk scores based on glycosylation patterns has potential clinical implications and immune-related value for breast cancer.

## Introduction

Breast cancer has reached the highest incidence in women’s cancer types, and its lethality has reached second place, followed by lung cancer (Sung et al., 2021). As a heterogeneous disease, breast cancer’s multiple subtypes represent different histological origins, prognoses, and therapeutic sensitivity (Perou et al., 2000; Cancer Genome Atlas Network, 2012; Curtis et al., 2012; Marusyk et al., 2012). The pathological markers estrogen receptor (ER), progesterone receptor (PR), and human epidermal growth factor 2 (HER2) stratified patients with various treatment selecting, such as hormonal therapy (e.g., Tamoxifen) and HER2-targeted therapy (e.g., Trastuzumab) (Goldhirsch et al., 2013). Of note, HER2 is characterized by poor prognosis and has multiple sites of N-glycosylation, whose presence is linked with function (Peiris et al., 2017). Subsequently, intrinsic molecular subtyping based on expression profile highlights the intricate complexity of this cancer type and the importance of genomic/transcriptomic analyses for prognostic prediction. PAM50 utilizes a 50 genes system that classifies breast cancer into luminal A, luminal B, HER2-enriched, and basal-like subtype that involves not only diverse biological processes but also has prognostic significance (Prat et al., 2012; Prat et al., 2015). The highly heterogeneous of breast cancer requires a strong need for more specific biomarkers and broader alternatives for personalized treatment. Meanwhile, efforts to classify established histological subtypes have been carried out, which identified at least four distinct subtypes of ER-negative and six triple-negative subtypes (Teschendorff et al., 2007; Lehmann et al., 2011). According to recent reports, researchers are seeking a multi-angle classification approach to identify diversified functional clustering and signatures, such as glycolysis (Zhang et al., 2020a; Jiang et al., 2021), autophagy (Zhang et al., 2020b; Jiang et al., 2022), ferroptosis (Wang et al., 2021), stemness (Li et al., 2020), and immune microenvironment (Shen et al., 2020). All these attempts allow us to make more defined and precise characterizations based on new parameters to drive the heterogeneity landscape of breast cancer and put forward new ideas in prognostic prediction and treatment in the future.

Glycosylation is defined as a biosynthetic enzymatic process characterized by the covalent attachment of single sugar or glycans to a wide range of target proteins (Pinho and Reis, 2015; Eichler, 2019). As a post-translational modification, they play an essential role in almost all aspects of the life processes of cells, such as cell cycle, proliferation, and aging (Mallard and Tiralongo, 2017; Gudelj et al., 2018; Gao et al., 2021). The glycosylation pattern is profoundly altered during tumorigenesis. Among them, O-glycan truncation, sialylation, fucosylation, and N-glycan branching are common types of glycosylation in cancer (Drake et al., 2015; Kölbl et al., 2015; Kudelka et al., 2015; Taniguchi and Kizuka, 2015), leading to the occurrence of malignant phenotypes such as cell adhesion, metastasis, epithelial–mesenchymal transitioning, and even the shifting of the tumor microenvironment (Günthert et al., 1991; Rabinovich and Toscano, 2009; Pinho et al., 2011; Paredes et al., 2012; Pinho et al., 2013). Researchers have also identified glycosylation-related molecules as biomarkers for cancer diagnosis and prognostics evaluation. For instance, prostate-specific antigen (PSA) in prostate cancer (Gilgunn et al., 2013), carcinoma antigen 125 (CA125/MUC16) in ovarian cancer (Zurawski et al., 1988), CA19-9 and carcinoembryonic antigen (CEA) in colon cancer (Goldstein and Mitchell, 2005), and aberrantly glycosylated MUC1 (also known as CA15-3) in breast cancer (Kumpulainen et al., 2002). More recent studies have mapped the histopathological orientation and tissue distribution of N-linked glycans in clinical breast cancer tissues (Scott et al., 2019a; Scott et al., 2019b), which deepen the understanding of the heterogeneity of breast cancer from the perspective of glycosylation.

Our study classified breast cancer samples from The Cancer Genome Atlas (TCGA) into three groups based on glycosylation-associated genes and then identified differentially expressed genes under different glycosylation patterns to construct a prognostic model. Finally, a model containing 23 risk signatures was built and performed favorable predicting efficacy in training and testing cohorts, and the evaluation of immune infiltration and immunotherapy response were analyzed as well.

## Results

### Classification of BRCA based on the glycosylation-related gene sets

**FIGURE 1:**
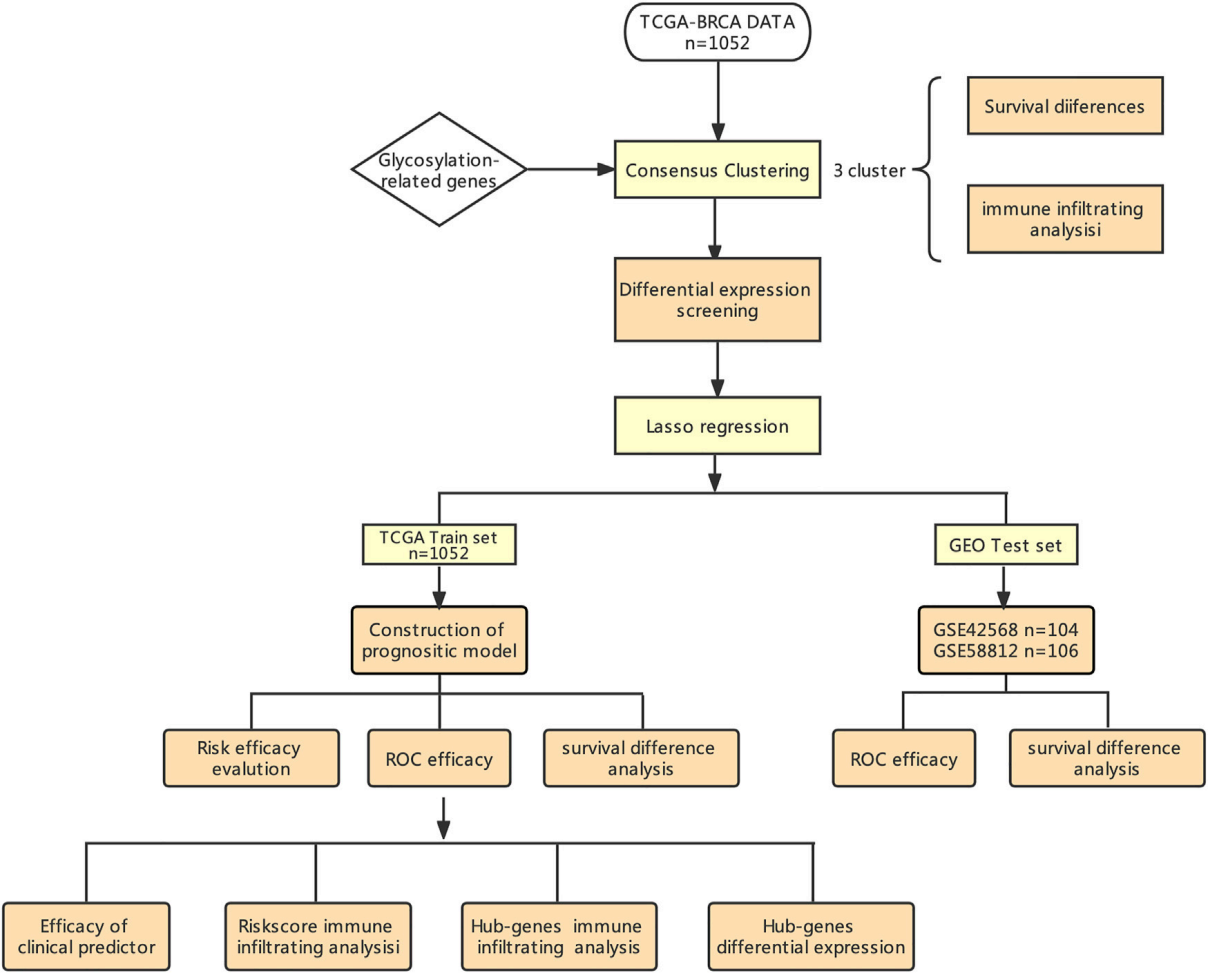
Workflow of our study design.

**FIGURE 2:**
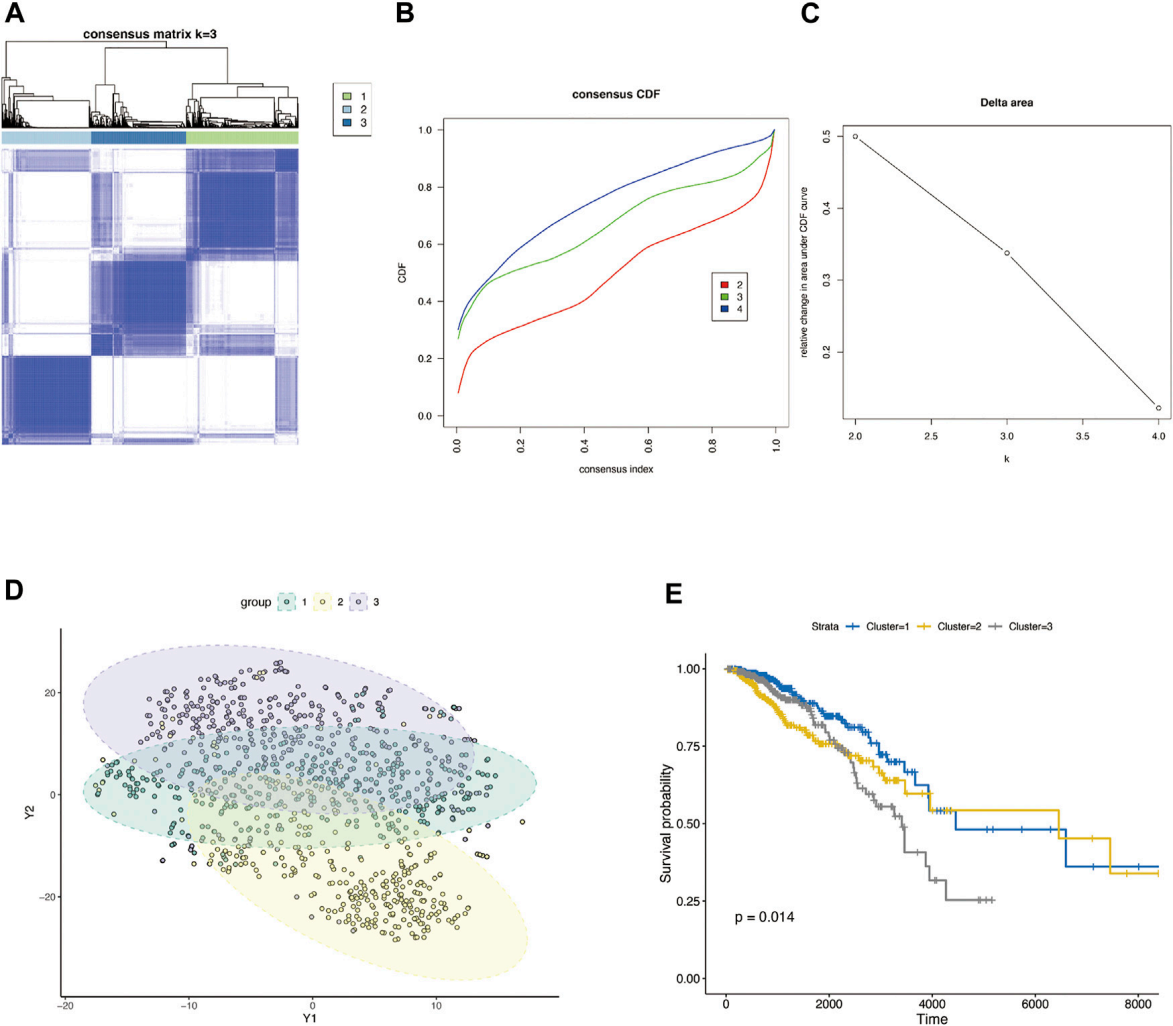
Consensus clustering classification of BRCA based on glycosylation-associated genes. **(A)** Optimal cluster distinction by consensus matrix (k = 3). **(B)** Empirical cumulative distribution function (CDF) plot displayed consensus distributions for each k. **(C)** Delta area plot. **(D)** T-SNE clustering of sample distributions based on glycosylation-related genes. **(E)** KM survival analysis of three glycosylation-based groups.

Figure 1 shows the workflow of our study. The TCGA column of Table 1. We classified TCGA-BRCA samples (*n* = 1,104) based on 179 glycosylation-related genes (GRGs) performed by consensus clustering analysis. Related clustering parameters are shown in Figures 2A–C, Supplementary Figure S1A, and Supplementary Figure S2A. Considering the complexity of grouping and the feasibility of subsequent analysis, we choose the optimal grouping when k = 3. Thus, we obtain three glycosylation-based clusters. We used t-SNE (Figure 2D) and PCA (Supplementary Figure S2B) dimensional reduction methods to observe that the samples had favorable overall differences under this grouping. Cluster 3 exhibited shorter overall survival (OS), indicating a poorer prognosis compared with clusters 1 and 2. (*p* < 0.05) (Figure 2E). In brief, this grouping method based on intracellular glycosylation status has specific differences in breast cancer samples and has substantial clinical value.

**TABLE 1:** Clinical information of TCGA, GSE42586, GSE58812.

	TCGA	GSE42568 PMID: 23740839	GSE58812 PMID: 25887482
Sample			
Tumor	1,109	104	107
Normal	113	17	0
Survival			
Dead	144	35	29
Alive	933	69	78
Age			
<60	575	59	64
≥60	502	45	43
Grade			
I	—	11	—
II	—	40	—
III	—	53	—
Stage			
I	179	11	—
II	609	40	—
III	246	53	—
IV	19	0	—
Unknown	24	0	—
Subtype			
Luminal A	497	—	—
Luminal B	197	—	—
Basal	171	—	—
Her2	77	—	—
Unknown	135	—	—
ER expression			
Positive	—	67	—
Negative	—	34	—

Her2, human epidermal growth factor receptor 2; ER, estrogen receptor.

### Screening of differentially expressed genes

We classified BRCA tumor samples into three clusters based on glycosylation patterns. Next, we screened the DEGs of these three clusters using the “Deseq2” R package. Supplementary Figures S2C–E show the PCA map and DEGs heatmap between the three clusters. Figure 4 shows the differential analysis volcano plot of group 1 to group 2 (Figure 4A), group 2 to group 3 (Figure 4B), and group 1 to group 3 (Figure 4C). We made a Venn diagram for the three groups of differential genes to show their overlap (Figure 4D). The genes contained in each unit are shown in Supplementary Table S1, and the genes that show differences under one grouping are included in the next analysis. Finally, 1915 DEGs (Supplementary Table S1) were obtained and used to construct a prognostic risk-scoring model.

### Immune characteristics of glycosylation-related groups

**FIGURE 3:**
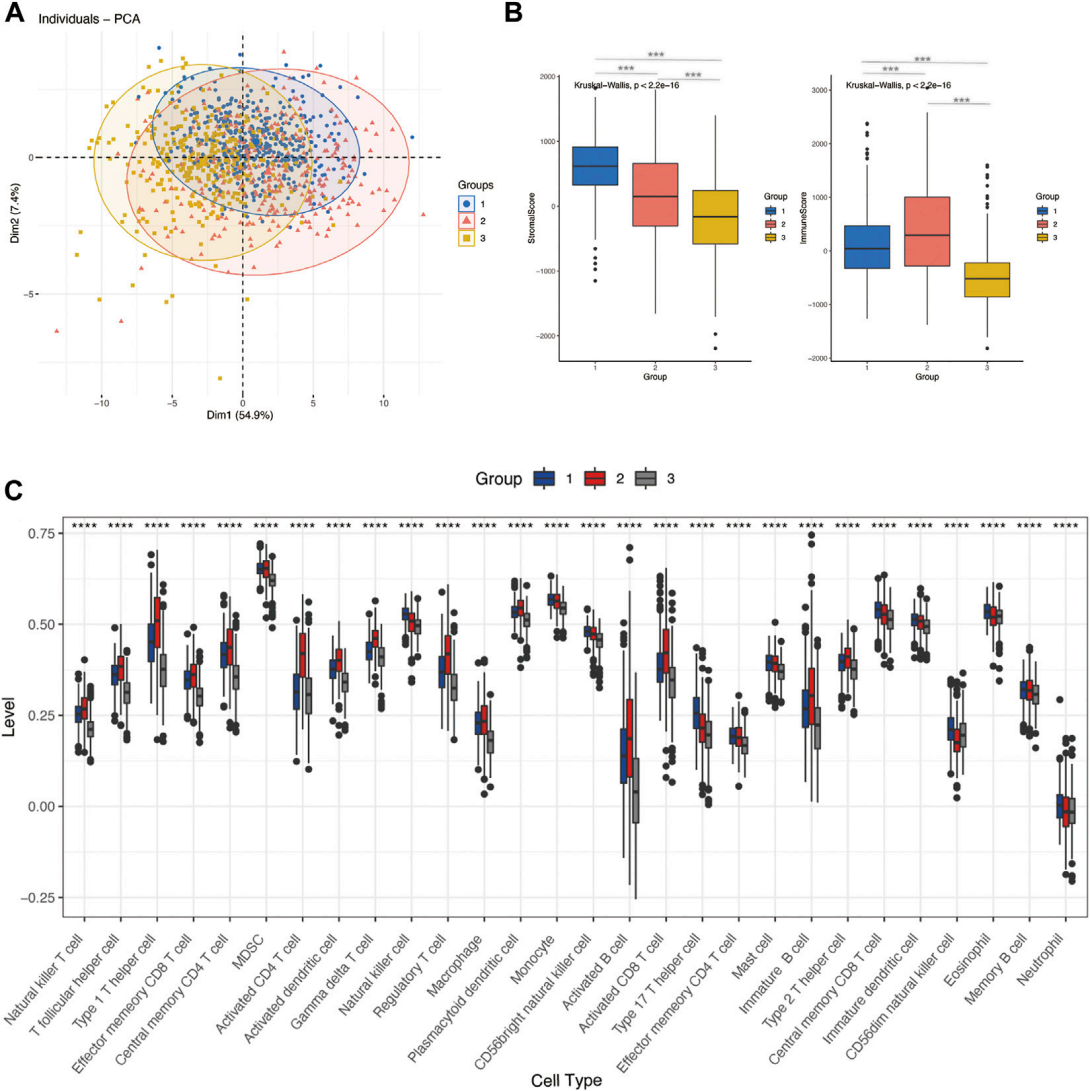
Differences in immune characteristics of glycosylation-based groups. **(A)** PCA clustering of sample distributions in immune signatures between three glycosylation-based groups. **(B)** Stomal score and the immune score of glycosylation-based groups (ESTIMATE algorithm). **(C)** Differences in 24 TME infiltration cells between glycosylation-based groups (ssGSEA) (****p < 0.0001).

To explore the correlation between glycosylation patterns and immune characteristics, we analyzed the immune correlates of the three clusters. Figures 3B and C show significant differences in the immune score, stomal score, and immune cell infiltration. Cluster 3 demonstrated the lowest immune and stomal score and the poorest immune cell infiltration. Cluster 2 had the highest immune score and modest stomal score, and the immune cell infiltration was also the most abundant. Cluster 1 had the mediocre immune score and highest stomal score, and the immune cell infiltration was modest.

### Construction and efficacy of risk-scoring model

**FIGURE 4:**
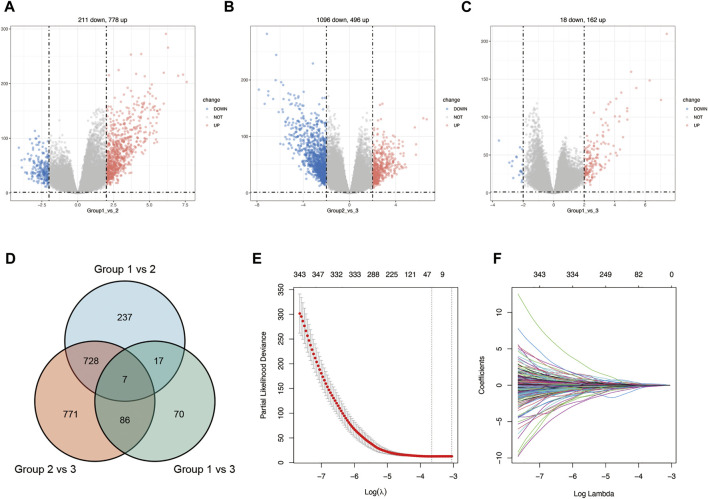
Construction of lasso regression model. Volcano plot of differentially expressed genes between cluster 1 vs. cluster 2 **(A)**, cluster 2 vs. cluster 3 **(B)**, and cluster 1 vs. cluster 3 **(C)** in BRCA. **(D)**. Venn diagram of differentially expressed genes between glycosylation-based groups. **(E)**. Cross-validation plot for the penalty term *λ* based on differentially expressed genes. Vertical bars represent acceptable maximum and minimum *λ* values with corresponding mean-squared error and the number of covariates. **(F)** Plots for lasso regression coefficients over different values of the penalty parameter *λ*.

**FIGURE 5:**
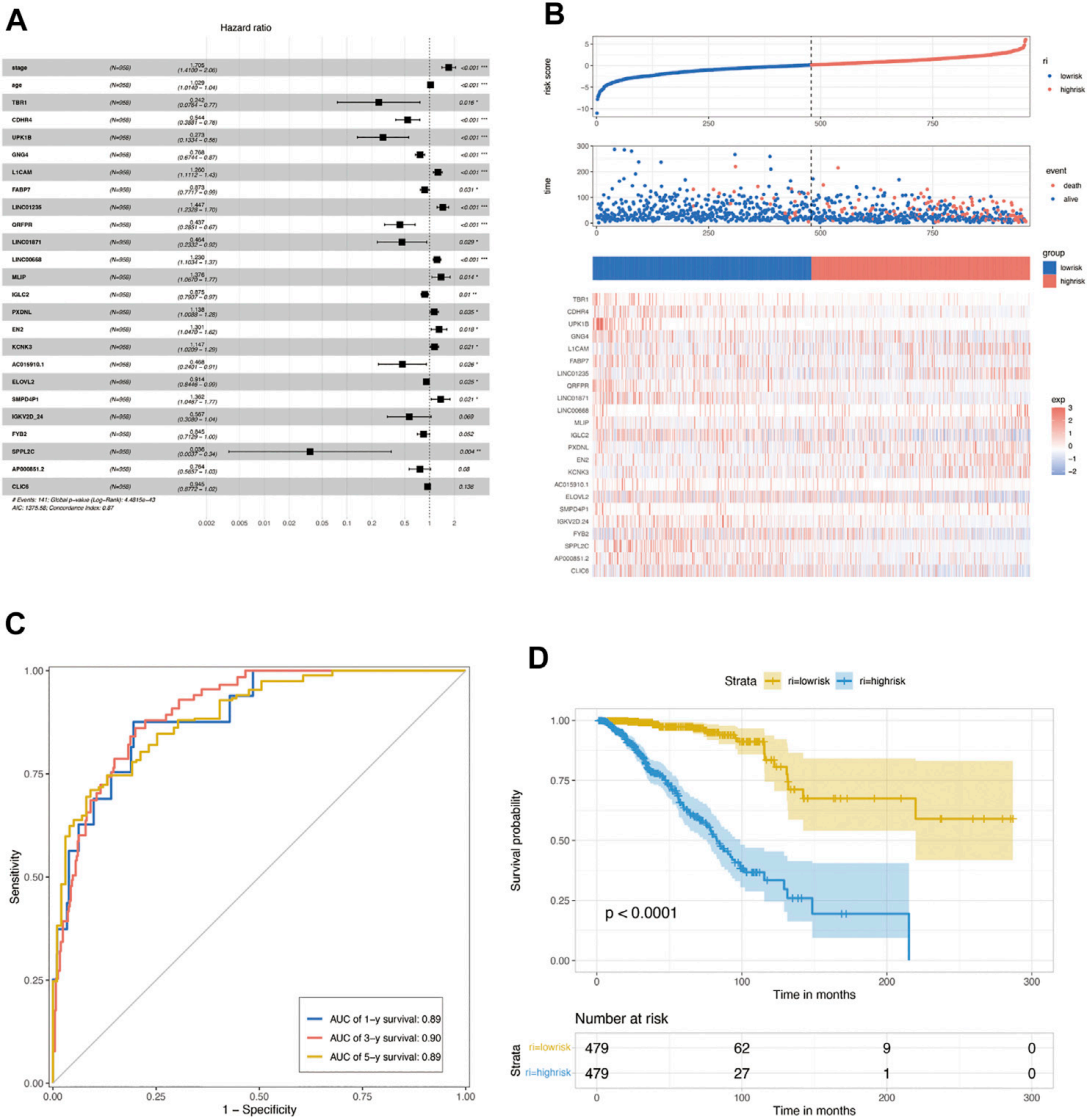
Construction of multivariate Cox regression. **(A)** Multivariate Cox proportional hazard model based on lasso de redundant gene set in the TCGA training set. **(B)** Proportion of deaths in the training set in high- and low-risk groups as risk score values increased. Top: red, high-risk; blue, low-risk. Middle: red, death; blue, alive. Bottom: hierarchical clustering of 14 key molecules between low- and high-risk groups. **(C)** COX risk score’s time-dependent ROC curves for 1, 3, and 5 years before death in the TCGA training set. In the training set, **(D)** Kaplan–Meier survival analyses for COX low- and high-risk groups. (*p* < 0.0001, log-rank test).

To further construct a prognostic risk-scoring model without redundant genes, we used lasso regression to narrow down the range of candidate genes. According to mean-square error (Figure 4E) and coefficients (Figure 4F), we opted for the former *λ* as it results in a better prediction efficiency than the latter *λ*. Then, we fitted a multivariate Cox proportional hazard model to develop more valuable integrated molecules in the training set. Patients’ age, stage, and 23 genes were included in this model, with a concordance index of 0.87 (Log-rank P: 4.48e-43) (Figure 5A). Figure 5B arranged the sample from low to high according to the risk score. The proportion of deaths increased as risk scores rose. The 23 key molecule expression is also shown at the bottom. Its area under the ROC curve (AUC) in 1, 3, and 5 years prior to death was 0.89, 0.90, and 0.89, respectively (Figure 5C). Kaplan–Meier (KM) analysis showed a significant difference in overall survival (*p* < 0.0001) (Figure 5D).

### Validating of risk-scoring model predicting efficacy

**FIGURE 6:**
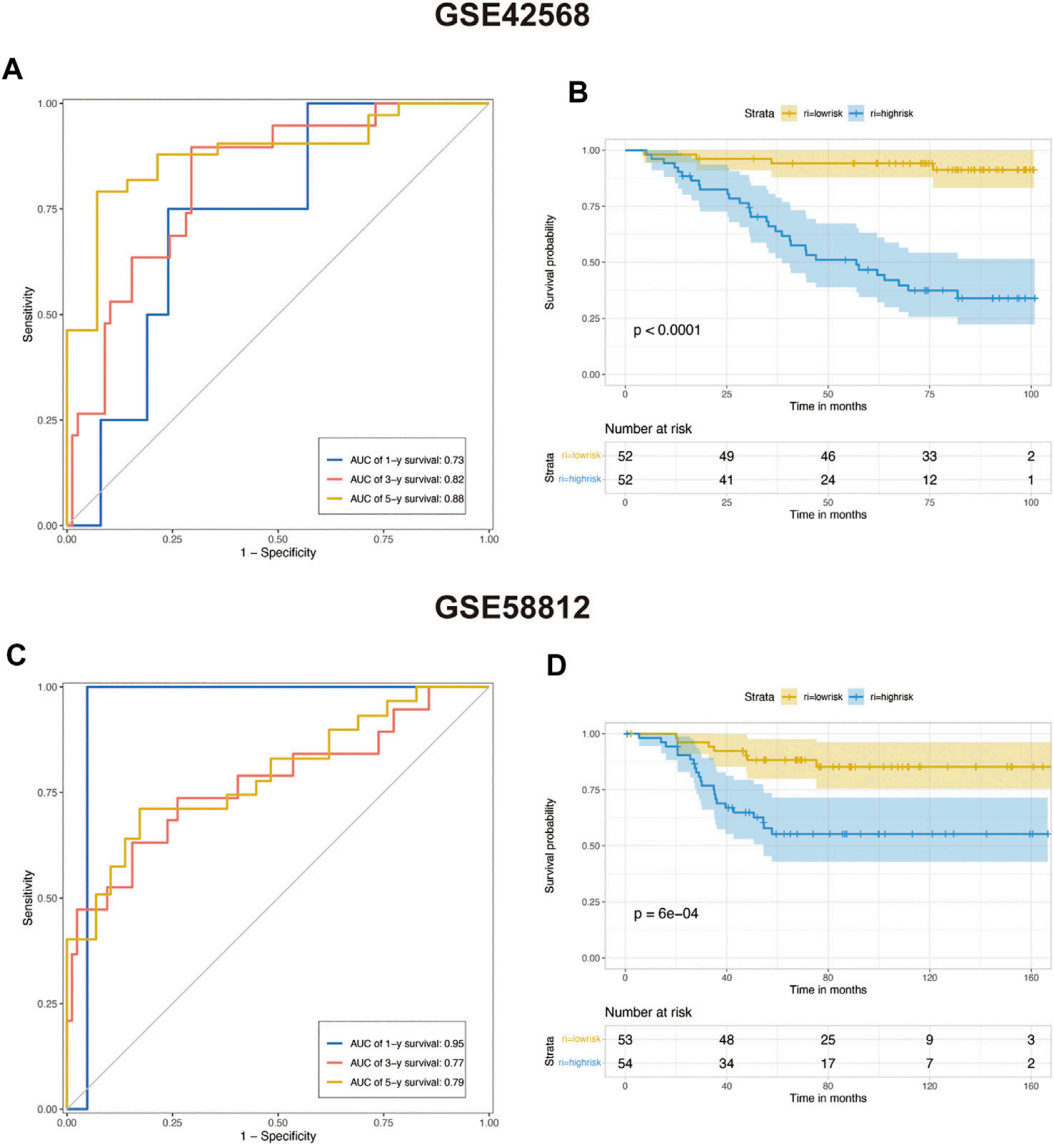
Predicting the efficacy of constructed multivariate Cox regression in the testing set. **(A)** COX risk score’s time-dependent ROC curves for 1, 3, and 5 years before death in testing cohort GSE42568. **(B)** Kaplan–Meier survival analyses for COX low- and high-risk groups in testing cohort GSE42568 (*p* < 0.0001, log-rank test). **(C)** COX risk score’s time-dependent ROC curves for 1, 3, and 5 years before death in testing cohort GSE58812. **(D)** Kaplan–Meier survival analyses for COX low- and high-risk groups in testing cohort GSE58812. (*p* < 0.0001, log-rank test).

We choose two breast cancer cohorts from GEO to validate the efficacy of this protistic model. The GSE42582 column of Table 1. In GSE42568 cohort, AUC in 1, 3, and 5 years prior to death was 0.73, 0.82, and 0.88, respectively (Figure 6A), and KM analysis presents a significant difference (*p* < 0.0001) (Figure 6B). The GSE58812 column of Table 1. In GSE58812 cohort, AUC in 1, 3, and 5 years prior to death was 0.95, 0.77, and 0.79, respectively (Figure 6C), and KM analysis presents a significant difference (p = 6e-04) (Figure 6D).

### Risk score related immune infiltration and immunotherapy evaluation

**FIGURE 7:**
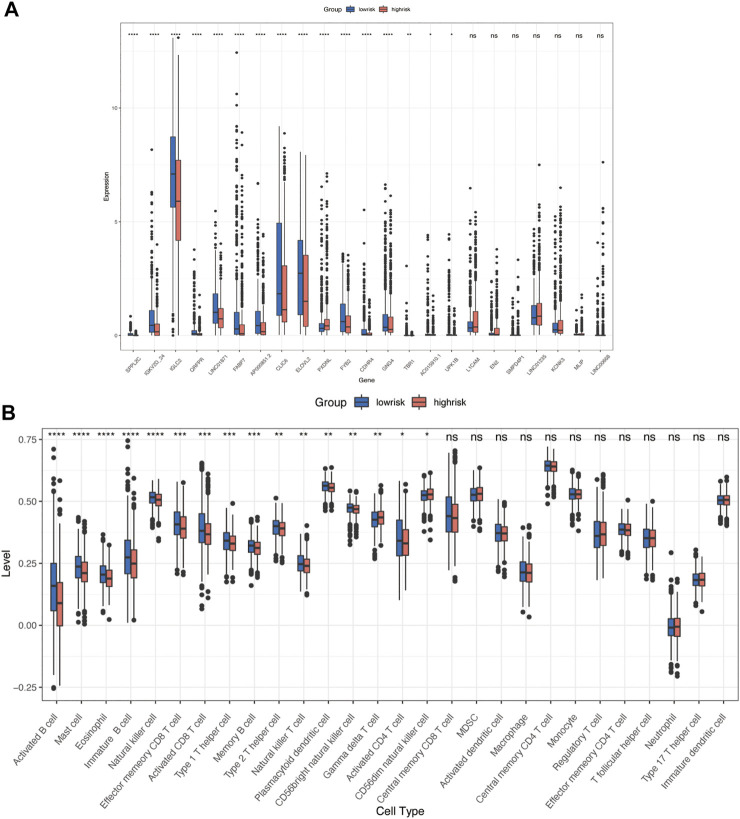
Immune characteristics in high- and low-risk groups. **(A)** Risk signatures expression in high- and low-risk groups. **(B)** Differences in 24 TME infiltration cells between high- and low-risk groups (ssGSEA) (*p < 0.05; **p < 0.01; ***p < 0.001, ****p < 0.0001).

**FIGURE 8:**
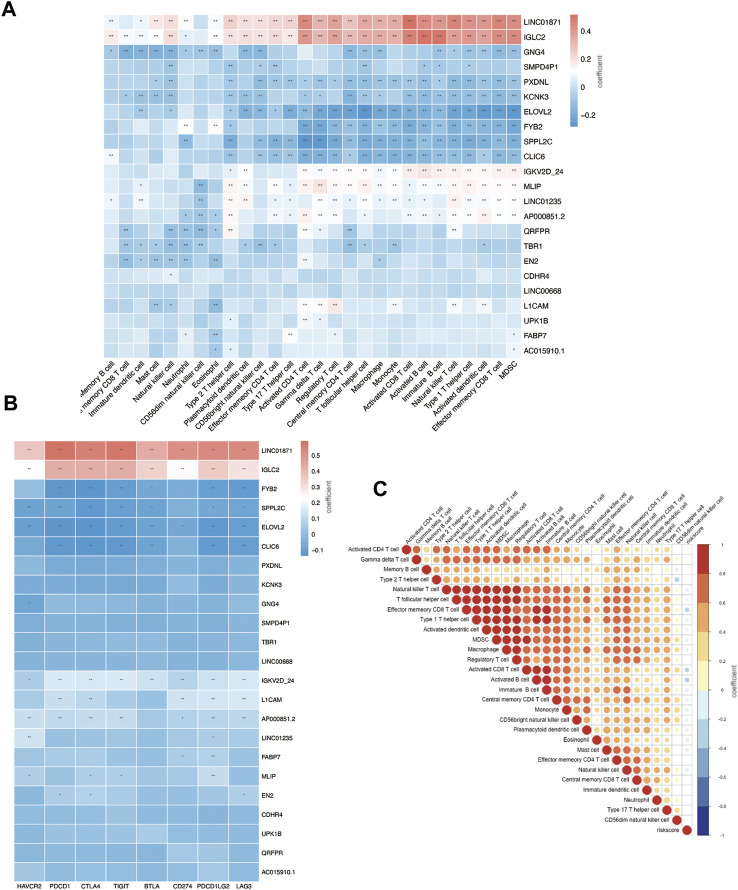
Immune infiltration status of prognostic signature. **(A)** Risk genes level in high- and low-risk groups. **(B)** Correlation between risk genes and checkpoint molecule expression. **(C)** Correlation between riskscore and immune infiltration.

**FIGURE 9:**
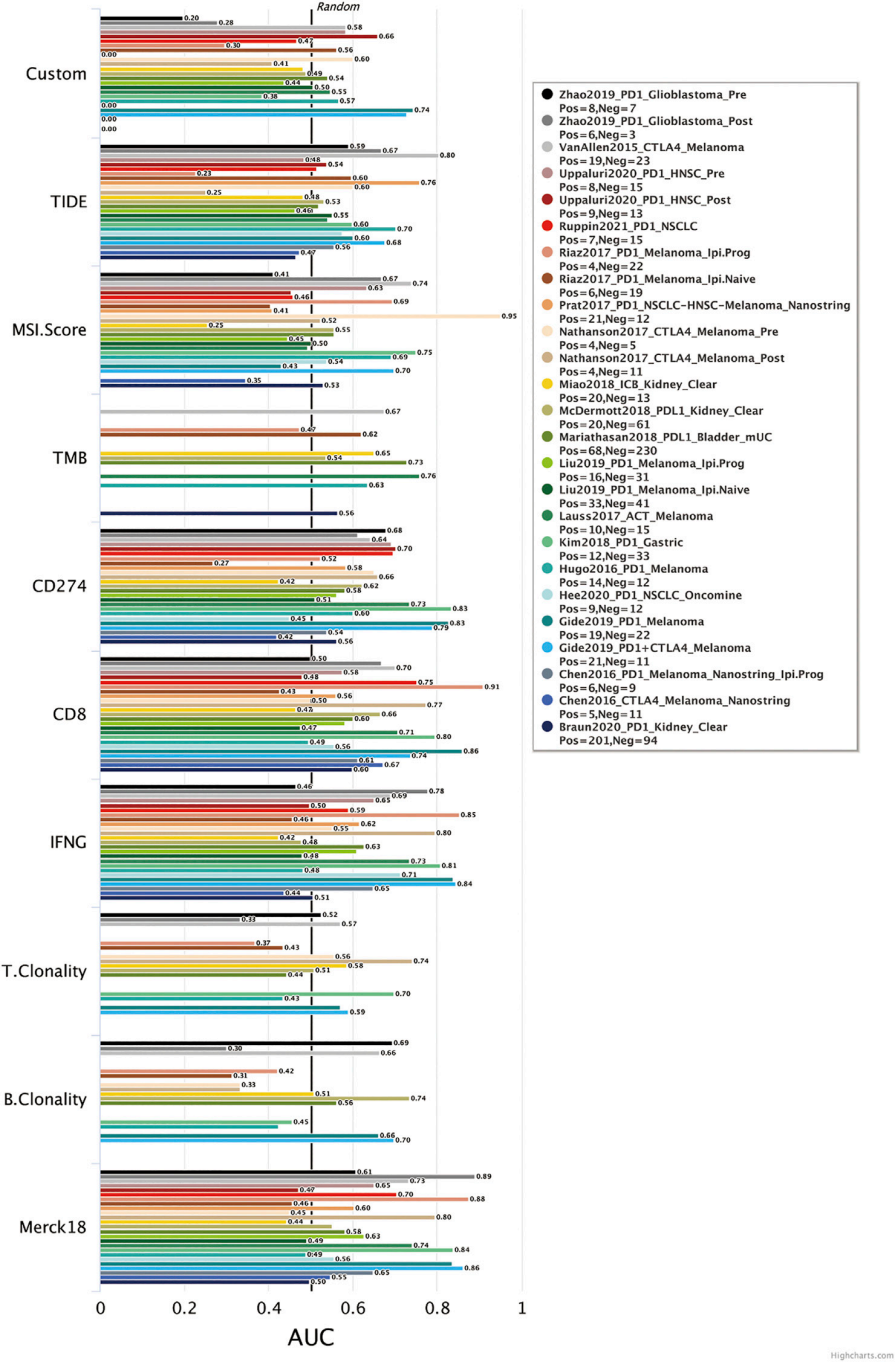
Biomarker evaluation from TIDE (Tumor Immune Dysfunction and Exclusion).

We calculated a risk score for each sample according to the expression levels and regression coefficients and divided the BRCA cohort into low- and high-risk groups by median. To better investigate the interactions between the risk score and the immune microenvironment, we performed the ESTIMATE algorithm and ssGSEA to evaluate the correlation between the prognostic model and immune infiltrating in BRCA patients. Supplementary figure S3A shows PCA clustering of immune signatures. The low-risk group demonstrates a higher immune score but no difference in the stomal score (Supplementary figure S3B). In terms of immune cell infiltration (Figures 7B, 8C), the risk score was slightly negatively correlated with immune cell level. The low-risk group represents a more significant fraction of activated B cells, eosinophils, mast cells, activated CD8^+^ T cells, natural killer cells, and effector memory CD8^+^ T cells but no difference in neutrophils, T follicular helper T cells, type 2 T helper cells, and type 17 T helper cells. Then, we used TIDE, an online tool, to evaluate immune checkpoint blockade (ICB) response for our screened signatures based on the TCGA and PRECOG cohorts. According to Figure 9, the gene set we input obtained almost equivalent area under the curve (AUC) as other predicting scores, especially CD274, CD8, IFNG, and Merck 18.

### 23 Gene signatures investigation

We further investigated the correlation between 23 gene signatures and immune cell infiltration. Compared with the low-risk group, the high-risk group harbors a low level of SPPL2C, IGKV2D-24, IGLC2, QRFPR, LINC01871, FABP7, AP000851.2, CLIC6, ILOVL2, FYB2, CDHR4, GNG4, TBR1, AC015910.1, and UPK1B and a high level of PXDNL. (Figure 7A). LINC01871, IGLC2, IGKV2D-24, MLIP, LINC01235, and AP000851.2 positively correlated with immune cell infiltration, and GNG4, PXDNL, KCNK3, ELOVL2, FYB2, SPPL2C, CLIC6 negatively correlated with immune cell levels. The main types of immune cells with different infiltrating were activated CD4^+^ T cells, activated CD4^+^ T cells, natural killer T cells, activated B cells, activated dendritic cells, and MDSC. (Figure 8A). In addition, LINC01871 and IGLC2 positively correlated with immune checkpoint molecules such as PD-1, PDL1, CTLA4, TIGIT, LAG3, and BTLA and negatively correlated with HAVCR2. FYB2, SPPL2C, ELOVL2, CLIC6, IGKV2D-24, L1CAM, and AP000851.2 (Figure 8B).

## Materials and methods

### Data collection

The Breast Cancer (BRCA) data from The Cancer Genome Atlas Program (TCGA) was accessed *via* UCSC Xena (http://xena.ucsc.edu/). A total of 179 genes encoding glycosylation enzymes, targets, and regulators were obtained from previous literature (Krushkal et al., 2017) and are listed in Supplementary Table S1.

### Consensus clustering analysis based on glycosylation-related genes

BRCA samples from TCGA were grouped into three clusters using the “ConsensusClusterPlus” (version1.60.0) R package (Wilkerson and Hayes, 2010) based on glycosylation-related genes (GRGs) (maxK = 4, innerLinkage = “complete”). “Fpkm” format was used for clustering analysis and “count” for difference analysis. Principal component analysis (PCA) and t-SNE were applied to assess sample clustering using the “FactoMineR” (version2.4) and Rtsne (version0.16) packages. “DESeq2” (version1.36.0) R package was used for screen differentially expressed genes (DEGs) between different clusters (|logFC| > 2, FDR <0.05).

### The prognostic risk-scoring model constructed through GRGs-based clusters

First, the most minor absolute shrinkage and selection operator (LASSO) removed redundant genes achieved using the “glmnet” (version 4.1-4) R package. Ten-fold cross-validation was used to select the penalty term, *λ*. The mean-squared error was computed for the test data to measure the fitted models’ predictive performance. Then, 38 genes (Supplementary Table S1) were obtained for prognostic Cox regression construction using the “My.stepwise” (version 0.1.0) package to establish the optimal model. Finally, the 23 retained genes were used for calculating risk scores according to the following formula:

$$Risk\;Score=\overset{n}{\underset{i=0}{\sum }}\left(Coefi^{*}xi\right),$$ (1)

where Coefi is the coefficient, and xi is the z-score-transformed relative expression value of each selected gene. The time-dependent receiver operating characteristic (ROC) curve evaluated each model’s sensitivity and specificity. The “survival” (version 3.3-1) R package was used, and the Kaplan–Meier (KM) overall survival curves between different clusters and risks were performed using the “survival” R package.

### Immune infiltrates analysis

The single-sample gene-set enrichment analysis (ssGSEA) was used to establish the relative abundance of 24 cell infiltration, which was analyzed using the “GSVA” (version 1.44.2) package. The ESTIMATE algorithm calculated stomal scores and immune scores of high- versus low-risk groups and different GRGs-based clusters. Immune checkpoint blockade (ICB) predicting evaluation performed by biomarker evaluation module from TIDE (Tumor Immune Dysfunction and Exclusion: harvard.edu)">http://tide.dfci.harvard.edu/) (harvard.edu)), a computational method to model tumor immune evasion and ICB response and resistance regulators.

### Hub-genes analysis

Immune Infiltrates differences of prognostic hub-genes were performed using ssGSEA, as mentioned earlier. Checkpoints correlation was analyzed using the ‘Hmisc’ (version 4.7-0) package. All the statistical significance sets as *p* < 0.05 with two-side. Data processing and visualization were performed using R version 4.1.2.

## Discussion

The role of glycocalyx–the extracellular carbohydrate coat, has been proposed in breast cancer occurrence and development since the 1950s (Aub et al., 1963). Then, it was noteworthy that plant lectin and carbohydrate motif binding proteins showed a higher affinity for malignant cells than normal cells in the 1960s (Remmele et al., 1986). By the 1980s, biochemists found that the enzyme-linked lectin binding assay could be used to predict tumor differentiation and therapeutic reactivity (Parodi et al., 1982). Shortly afterward, it was widely accepted that glycosylation status alteration could be used as biomarkers for breast cancer prognosis and tumor burden (Springer, 1997; Lin et al., 2002; Duffy et al., 2010). Given the heterogeneity of breast cancer, more recent studies have mapped the histopathological orientation and tissue distribution of glycosylated modifications in clinical breast cancer samples. So far, the influentially changed landscape of glycosylation processes in breast cancer is vividly portrayed.

We obtained a set of glycosylation-related genes containing 181 molecules from previous pieces of literature, including glycosylation pathways, genes encoding glycosylation targets or regulators, and members of cancer pathways affected by glycosylation (Supplementary Table S1) (Krushkal et al., 2017). In our study, TCGA-BRCA tumor samples were divided into three groups. We can consider three different glycosylated states based on these glycosylation-related genes by using consensus clustering analysis. There were significant differences in the expression patterns of glycosylated genes between them, and the survival analysis also reflected the difference in survival time under different glycosylated states (Figures 2D and E). It is well-documented that an altered “glycan coat” is a distinct hallmark of cancer.

Given that immune cells express a large variety of lectin (glycan-binding receptors), they recognize glycans on the tumor cell. Those immune cells can sense and respond to changes in the glycan signature of their environment. This often leads to tumor immune escape and immunomodulation. Therefore, the glycosylation-related signatures could affect tumor-immune cells’ connections within the tumor microenvironment (Rodríguez et al., 2018; Lopes et al., 2021). In addition, a variety of recruited stomal components–transformed parenchyma and the associated stroma–are involved in tumor progression and response to treatment (Arneth, 2019; Hanahan, 2022). We further analyzed the immune characteristics of glycosylation-based groups. According to our results, group 3 demonstrated the lowest immune and stomal score and the poorest immune cell infiltration; group 2 had the highest immune score and modest stomal score, and the immune cell infiltration was also the most abundant. This indicates that group 2 tends to the glycosylation pattern of immune cells, group 1 of stromal cells, and group 3 of malignant cells (Figures 3A–C). In combination with the survival analysis of Figure 2E, we were surprised to find that in terms of glycosylation pattern, the glycosylation mode of tumor cells and immune cells did not show any difference in patient survival, while the glycosylation of stromal cells may have a significant impact on patients’ survival. In future explorations of tumor microenvironment glycosylation, focusing on stromal cells may be a more effective research direction. These results prove that the classification based on glycosylation is meaningful and effective, which helps us to understand the heterogeneity of breast cancer from another perspective. However, at present, the classification samples are limited. Increasing the sample size will help formulate a more stable grouping method and hopefully be applied to clinical prognosis and prediction.

The change of glycosylation pattern in tumor cells and immune microenvironment will affect the expression of other critical genes and make their corresponding bioprocesses abnormal, thus, inducing the transformation of malignant phenotypes, such as proliferation, epithelial–mesenchymal transition, and apoptosis resistance. To identify the prognostic genes influenced by glycosylation processes, we screened the DEGs of these three groups and constructed a predictive risk model through lasso and Cox regression calculation. The final prognostic model containing 23 key molecules achieved exciting performance both in the TCGA training set and testing set GSE42568 and GSE58812 (Figures 5C and D, Figure 6). Using the model algorithm, we calculated a risk score and divided the sample into high- and low-risk groups by the median. This risk score also showed a significant difference in predicting overall clinical survival and immune infiltration (Figures 7B, 8C). Great achievement has been obtained in ICB-based immunotherapies (Chen et al., 2020). In order to obtain better clinical remission and fewer immune-related adverse events, researchers are committed to developing biomarkers to screen an effective population accurately. The reported measures that can be used to predict the efficacy of ICI therapy include immune cell infiltration (Cogdill et al., 2017), protein expressions such as PD-L1 (Teng et al., 2015), mutations and neoantigens (Mcgranahan et al., 2016), and genetic and epigenetic characteristics (Ascierto et al., 2012). On the TIDE prediction website, our gene set shows a favorable performance compared with the existing evaluation methods (Figure 9), which proves that our model has practical proficiency and value for further exploration and improvement in immunotherapy prediction.

Then, we move on to several single prognostic genes. LINC01871 significantly lower expression in the high-risk group and positively correlated with most of the immune cell infiltration (Figure 8). This suggests that LINC01871 may play a protective role in breast cancer. According to a recent review of the literature, LINC01871 has been identified by several studies in breast cancer through bioinformatic measurement involving the cellular phenotype of autophagy (Li et al., 2021; Wu et al., 2021; Jiang et al., 2022; Luo et al., 2022), stemness (Li et al., 2020), immune response (Ma et al., 2020; Mathias et al., 2021), ferroptosis (Xu et al., 2021), and lipid metabolism (Shi et al., 2022). IGLC2 has a similar expression and functional pattern to LINC01871 in our study (Figure 8). Chang et al. (2021) found in a study of triple-negative breast cancer (TNBC) cohort that a high expression of IGLC2 was related to a favorable prognosis for TNBC patients, which is consistent with our results. In addition, IGLC2 is linked with the proliferation, migration, and invasion of MDA-MB-231 cells. Pathway enrichment analysis showed that IGLC2 is related to the extracellular matrix–receptor interaction (Chang et al., 2021). All these features make IGLC2 have the potential to be a biomarker to predict prognosis, even for identifying breast cancer patients who can benefit the most from immune checkpoint blockade treatment. ELOVL2 is another prognostic signature in our results. Studies have shown that long noncoding RNA on its antisense chain (ELOVL2-AS1) correlates with breast cancer prognosis. The predictive efficacy of ELOVL2 needs to be verified in a larger sample size, and its mediated cell function also needs to be further explored.

## Data Availability

The datasets presented in this study can be found in online repositories. The names of the repository/repositories and accession number(s) can be found in the article/Supplementary Material.
