# Supplementary figures and images for: Identification of a prognostic risk-scoring model and risk signatures based on glycosylation-associated cluster in breast cancer
Source: Front Genet. 2022 Oct 20;13:960567. doi: 10.3389/fgene.2022.960567 (PMC9630632; doi:10.3389/fgene.2022.960567)

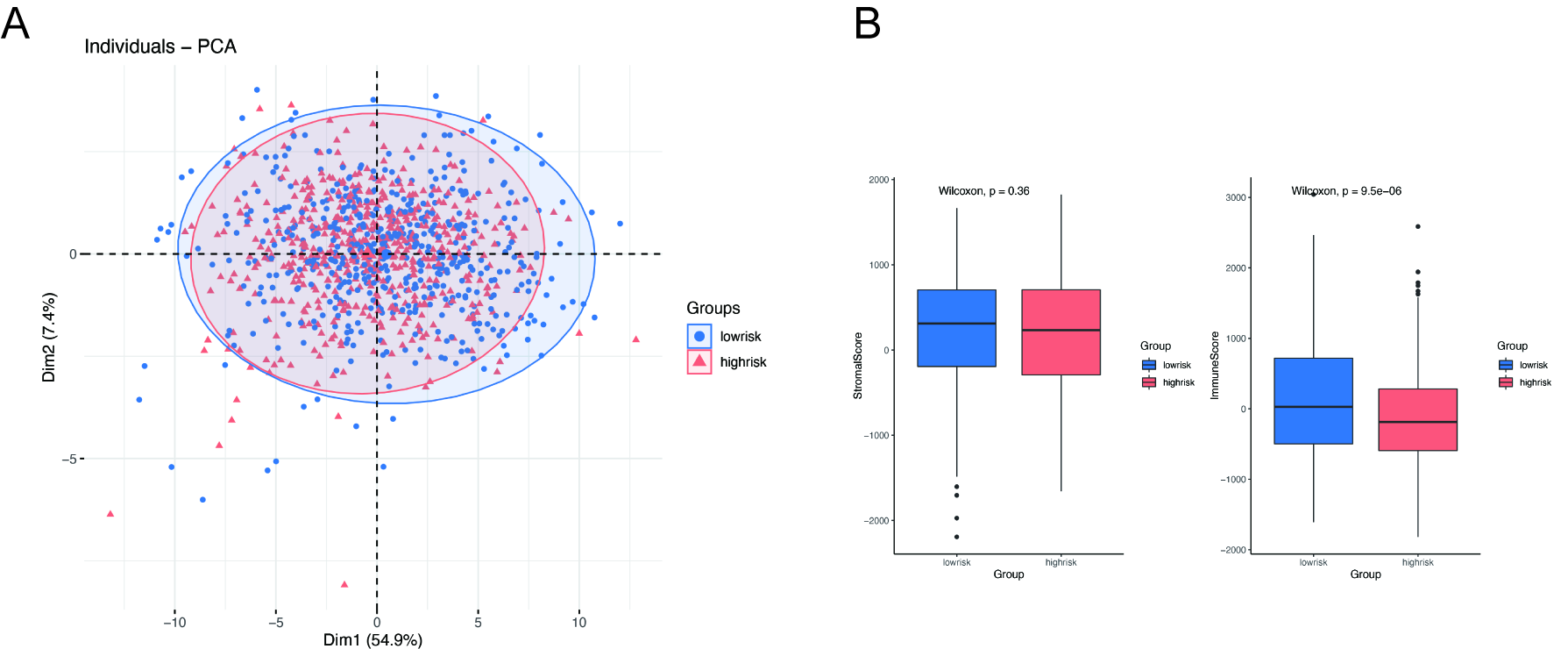

Supplement: Supplementary file 1 [file Image3.TIF]

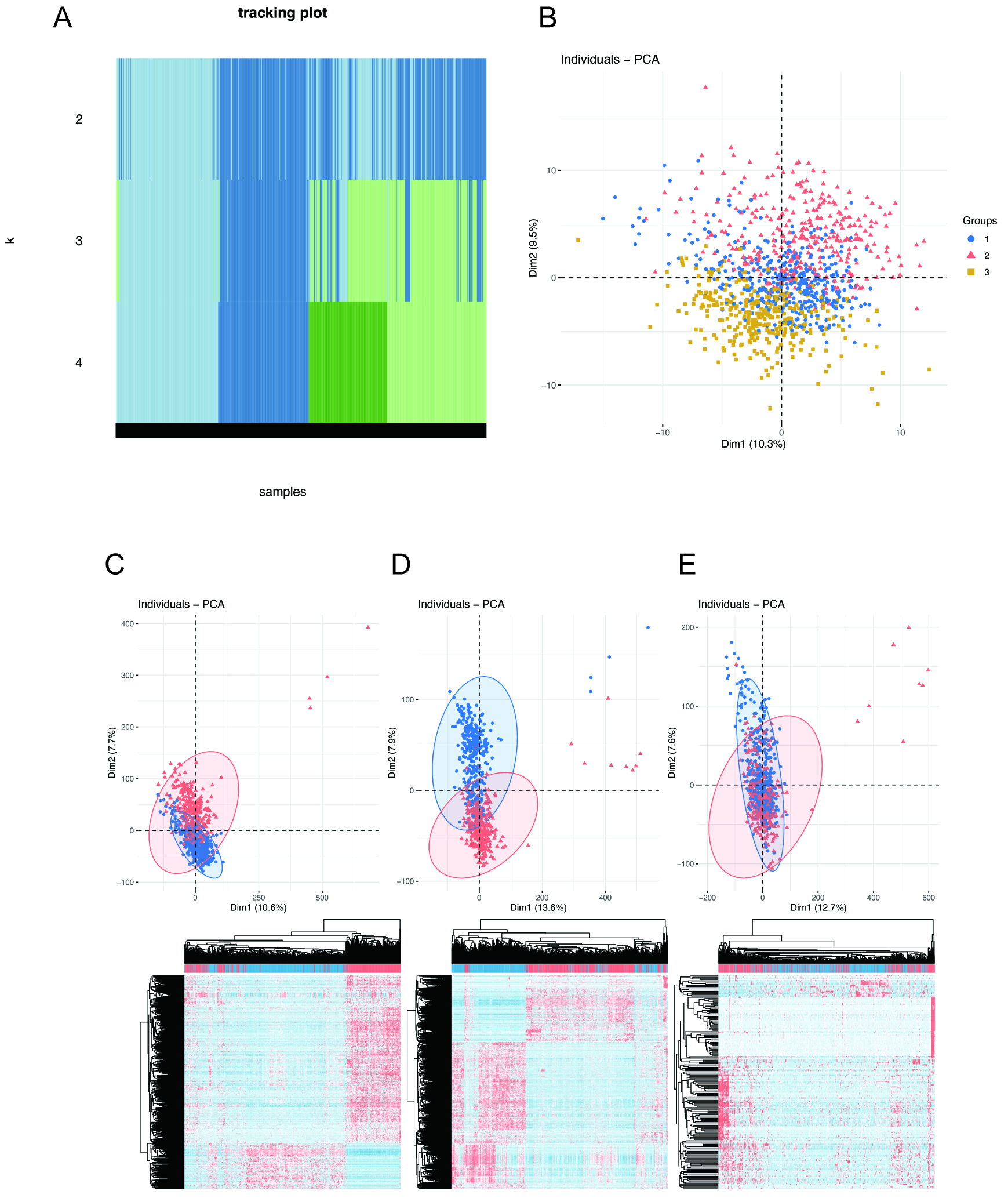

Supplement: Supplementary file 2 [file Image2.TIF]

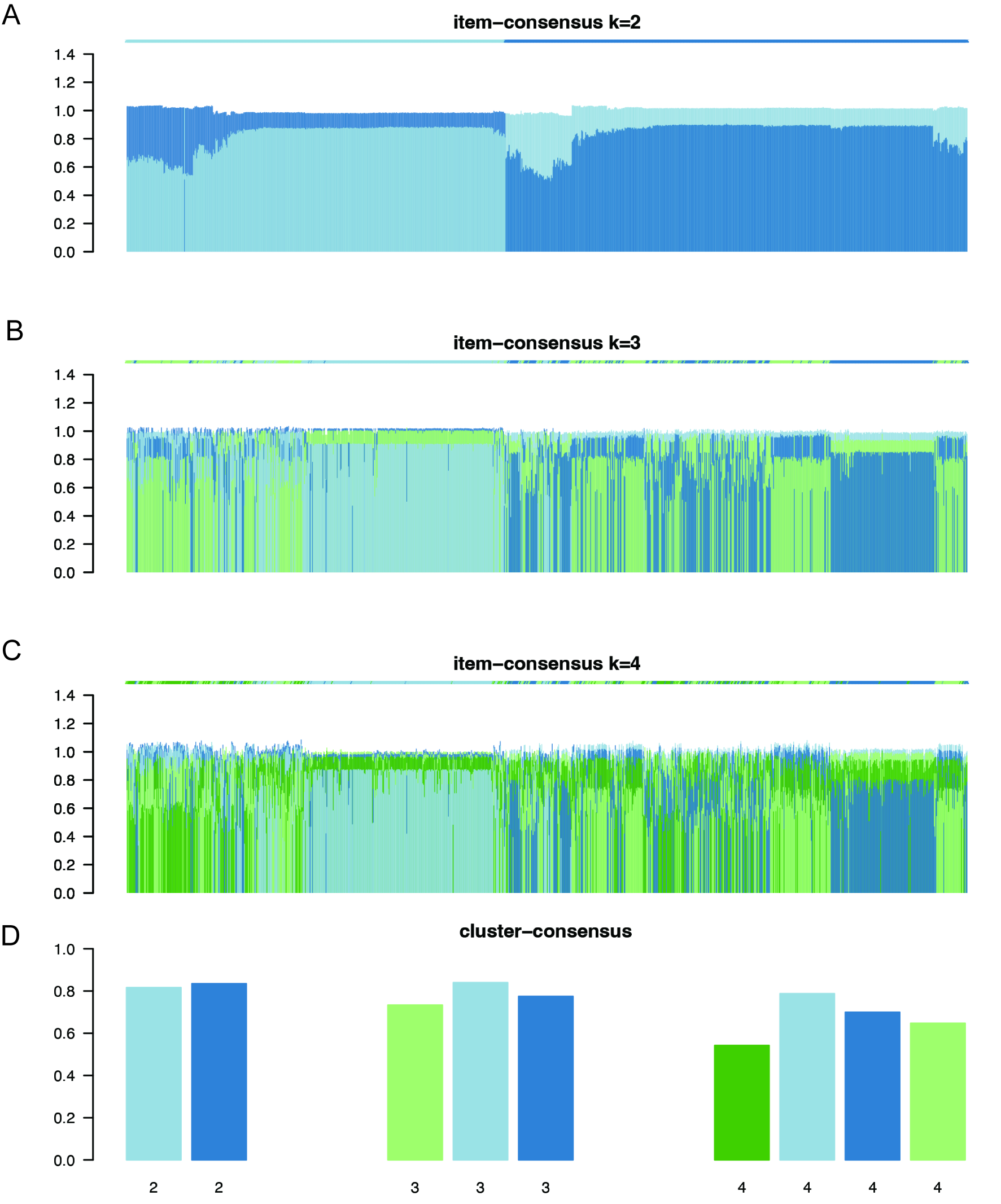

Supplement: Supplementary file 3 [file Image1.TIF]
